# Role of LncRNAs in the Pathogenesis of Coronary Artery Disease

**DOI:** 10.31083/j.rcm2404096

**Published:** 2023-03-23

**Authors:** Xinyu Wu, Jingru Li, Guihu Sun, Jun Yang, Yunzhu Peng, Xiangfeng Bai, Luqiao Wang

**Affiliations:** ^1^Department of Cardiology, The First Affiliated Hospital of Kunming Medical University, 650032 Kunming, Yunnan, China; ^2^Department of Cardiac Surgery, The First Affiliated Hospital of Kunming Medical University, 650032 Kunming, Yunnan, China

**Keywords:** lncRNA, coronary artery disease, endothelial cells, cardiomyocytes, vascular smooth muscle cells, cardiac fibroblasts, diagnostic biomarkers

## Abstract

Coronary artery disease (CAD), caused by coronary artery occlusion, is a common cardiovascular disease worldwide. Long non-coding RNAs (lncRNAs) are implicated in the regulation of endothelial cell injury, angiogenesis, plaque formation, and other pathological mechanisms in CAD by acting on different cell types. Some lncRNAs are significantly upregulated in CAD patients; however, other lncRNAs are significantly downregulated. Differential expression of lncRNAs in CAD patients enables them to be exploited as potential biomarkers to evaluate disease progression and diagnosis/prognosis in CAD patients. In this study, we reviewed the role of lncRNAs in the development of different clinical subtypes of CAD.

## 1. Introduction

Coronary artery disease (CAD) is one of the most deadly diseases worldwide [1]. Clinically, CAD is divided into stable angina pectoris, unstable angina pectoris, and acute myocardial infarction (AMI). AMI is associated with inflammation, fibrosis, and angiogenesis, and can lead to heart failure in severe cases [2]. The typical mechanism of AMI is the formation and progression of intracoronary thrombus, which in turn produces varying degrees of thrombotic stenosis/occlusion, ultimately leading to myocardial necrosis and the formation of circumferential myocardial scarring in the area of coronary artery supplied [3, 4]. Molecular mechanisms such as mitochondrial dysfunction, inflammation, oxidative stress, and excessive fibrosis can lead to adverse cardiac remodeling in the late stage of an AMI, which results in increased morbidity and mortality [5]. Studies have found that several long non-coding RNAs (lncRNAs) are abnormally expressed in biological samples extracted from CAD patients. LncRNAs can regulate pathological mechanisms and disease progression through different target genes or signaling pathways, making them essential biomarkers [6]. LncRNAs, a non-coding RNA larger than 200 nucleotides, play a biological role in CAD by acting on downstream target molecules such as microRNAs (miRNAs, miRs), mRNAs, or transcription factors [7, 8]. Most lncRNAs act as competing endogenous RNAs (ceRNAs) and regulate the expression and activation of downstream mRNAs by competitive binding with miRNAs, thus affecting the cardiovascular system’s biological functions, such as cell proliferation, migration, and apoptosis. Therefore, lncRNAs may have an important regulatory role in the pathophysiology of CAD [9]. The lncRNA-miRNA-mRNA pathway is a classic ceRNA mechanism. Learning more about lncRNAs and their role in CAD will help to develop new treatment and diagnostic methods for CAD patients. In this review, we aim to explore the biological role of lncRNAs in various subtypes of CAD.

## 2. LncRNAs in CAD

The mechanism and function of lncRNAs in CAD have been widely studied in various cell types, most extensively in endothelial cells. Wang *et al*. [10] demonstrated that lncRNA *p21* acts on *miR-221* through the ceRNA mechanism, forming the miR-221/SIRT1/Pcsk9 axis. LncRNA *p21* overexpression can inhibit endothelial cell apoptosis and promote endothelial cell proliferation, migration, and tube formation, thus reducing subcutaneous lipid deposition to prevent the progression of atherosclerosis (AS). LncRNA has been downregulated in AS patients and AS mouse models. Similarly, compared with healthy subjects, the expression level of lncRNA *TONSL-AS1* in the plasma of CAD patients is also downregulated. Forced overexpression of this lncRNA in primary human coronary artery endothelial cells has been shown to promote proliferation and inhibit apoptosis by upregulating B-cell lymphoma-2 (BCL-2) expression levels through the negative regulation of *miR-197* in these cells [11]. Furthermore, Kai *et al*. [12] have reported that lncRNA *NORAD* (*NORAD*, non-coding RNA activated by DNA damage) expression levels were significantly upregulated in CAD patients and oxidized low-density lipoprotein (ox-LDL)-treated human umbilical vein endothelial cells (HUVECs). LncRNA *NORAD* is closely related to the occurrence and development of AS. *NORAD* recruits HDAC6 by enriching for FUS (FUS RNA binding protein), then HDAC6 binds to the promoter region of the *VEGF *gene, enhancing the level of H3K9ac deacetylation in this region and thereby inhibiting *VEGF* gene transcription. Li *et al*. [13] have shown upregulation of lncRNA *uc003pxg.1* and downregulation of *miR-25-5p* in peripheral blood mononuclear cells from CAD patients. LncRNA *uc003pxg.1* has been shown to promote the proliferation and migration of HUVECs by upregulating cyclinD1 and CDK6 via negatively downregulating *miR-25-5p* in an *in vivo* study. Interestingly, two different transcripts of lncRNA *ANRIL* exert diametrically opposing effects on CAD endothelial cells, which highlights the molecular characteristics of lncRNAs and their diverse biological roles [14]. It is unknown whether other CAD-related lncRNAs have multiple transcripts and different functions. Table 1 (Ref. [10, 11, 12, 13, 15, 16, 17, 18, 19, 20, 21, 22]) shows the functional roles of lncRNAs, as assessed in endothelial cells, in the occurrence and development of CAD.

**Table 1.** **CAD-related lncRNAs whose function has been assessed in endothelial cells**.

LncRNA	Sample	Expression pattern	Assessed cell lines	Gene/Protein interactions	Signaling pathway	Function	Ref
*p21*	Peripheral blood samples from 25 patients with AS and 18 health controls	Downregulated	HAECs	*miR-221*	-	Inhibits HAEC proliferation, migration, and tube formation by decreasing *miR-221*	[10]
*TONSL-AS1*	Peripheral blood samples from 60 patients with CAD and 60 health controls	Downregulated	HCAECs	*miR-197*	-	Increases migration and suppress apoptosis of HCAECs through regulating miR-197/BCL2 axis	[11]
*NORAD*	Peripheral blood mononuclear cells samples from 15 patients with CAD and 15 health controls	Upregulated	HUVECs	FUS	-	Its knockdown attenuates vascular endothelial injury through increasing *VEGF* gene transcription via enhancing H3K9 deacetylation by recruiting HDAC6	[12]
*uc003pxg.1*	Peripheral blood mononuclear cells samples from 80 patients with CAD and 80 health controls	Upregulated	HUVECs	*miR-25-5p*	-	Its knockdown attenuates migration and proliferation of human umbilical vascular endothelial cells through increasing *miR-25-5p*	[13]
*HIF1A*	Peripheral blood samples from 80 ApoE^-/-^ C57BL/6J male mice aged 4–6 weeks old and weighed 16–21 g (AS group included 60 mice and the sham group included 20 mice)	Upregulated	HCAECs/ECs	USF1	-	Its knockdown inhibits the ox-LDL induced atherosclerotic inflammation in ECs and HCAECs by downregulating ATF2 via binding USF1	[15]
*KCNC3-3:1*	Peripheral blood mononuclear cells samples from 93 patients with CAD and 48 health controls	Upregulated	HUVEC	-	JAK1/STAT3 signaling pathway	Its knockdown attenuates human umbilical vascular endothelial cell migration by suppressing the JAK1/STAT3 signaling pathway	[16]
*THRIL*	Peripheral blood samples from 20 patients with CAD and 20 health controls	Upregulated	EPCs	FUS	AKT signaling pathway	Its knockdown attenuates apoptosis and autophagy by targeting FUS and activating AKT signaling pathway	[17]
*NEAT1*	Peripheral blood samples from 40 patients with CAD and 40 health controls	Upregulated	HCAECs	*miR-140-3p*	-	Promotes apoptosis of HCAEC through regulating NEAT1/miR-140-3p/MAPK1 axis	[18]
*ANRIL*	Patients with CAD between January 2018 and July 2018 and sex-matched health control	Upregulated	HUVECs	*let-7b*	TGF-βR1/Smad signaling pathway	Its knockdown inhibits inflammation response and regulates endothelial	[19]
						Dysfunction by inhibiting *let‐7* and targeting TGF‐βR1/Smad signaling pathway	
*AK136714*	Male C57BL/6J mice and C57BL/6J, ApoE^-/-^ mice	Upregulated	HUVECs	HuR and FOXO3	-	Its knockdown inhibits ECs apoptosis and inflammatory responses by binding to FOXO3	[20]
*EZR-AS1*	Blood samples from 35 patients (24 men and 11 women; 50–75 years of age) with CHD and 38 individuals without CHD (22 men and 16 women; 50–75 years of age)	Upregulated	-	SMYD3	-	Downregulation of *EZR-AS1* inhibits the proliferation, migration, and apoptosis of HUVECs via SMYD3	[21]
*CASC11*	Plasma samples from 82 patients with CAD and 82 healthy controls	Downregulated	HCAECs	-	-	-	[22]

Abbreviations: HAECs, human aortic endothelial cells; EPCs, endothelial progenitor cells; HUVECs, human umbilical vein endothelial cells; HCAECs, human coronary artery endothelial cells; ECs, endothelial cells; CHD, coronary heart disease.

Kang *et al*. [23] have reported overexpression of lncRNA *AL355711* in atherosclerotic plaques and animal models of AS, and matrix metalloproteinase-3 (MMP3) has been associated with vascular smooth muscle cell (VSMC) migration in cardiovascular disease. Furthermore, knockdown of lncRNA *AL355711* has also been demonstrated to inhibit AS progression by regulating VSMC migration through the ABCG1/MMP3 pathway. Upregulation of lncRNA *CDKN2B-AS1* has been proven to upregulate *PTPN7* through competitive binding with *miR-126-5p*, attenuating VSMC proliferation and promoting apoptosis. However, this lncRNA showed decreased expression in ox-LDL-induced VSMC models and serum samples from CAD patients. Transfection of pcDNA-CDKN2B-AS1 into ox-LDL-induced VSMCs has been demonstrated to result in the upregulation of lncRNA *CDKN2B-AS1*, which may play an important biological role; however, these findings have not been studied *in vivo* [24]. In addition, lncRNA *Kcnq1ot1* has been found to stimulate the expression of *Tead1* by competitively acting on *miR-466k* and *miR-466i-5p*, thus promoting injury and apoptosis of cardiomyocytes. However, this specific mechanism still needs to be clarified in further studies [25].

Knockdown of lncRNA *Mirt2* has been shown to exacerbate hypoxia/reoxygenation-induced H9C2 myocardial cell damage and promote the progression of ischemic myocardial infarction in AMI rat models, while there was no significant effect on the normal or sham group. Upon further study of this mechanism, it was found that overexpression of *Mirt2* could upregulate *PDK1* levels through the negative regulation of *miR-764* and inhibit myocardial apoptosis and injury. Thus, a signaling axis of lncRNA Mirt2/miR-764/PDK1 protecting cardiomyocytes was formed [26]. Table 2 (Ref. [15, 24, 25, 26, 27, 28, 29, 30, 31, 32, 33, 34]) lists those CAD-related lncRNAs whose functions have been evaluated in cardiomyocytes or VSMCs. Some studies have shown that vascular aging is a specific risk factor for CAD. VSMCs play an important role in the pathological processes of vascular remodeling and stiffness associated with vascular aging [35, 36]. Therefore, there may be some potential correlations between the effects of lncRNAs on VSMC functions and vascular aging that have yet to be explored.

**Table 2.** **CAD-related lncRNAs whose function has been assessed in myocardial cells or vascular smooth muscle cells**.

LncRNA	Sample	Expression pattern	Assessed cell lines	Gene/Protein interactions	Signaling pathway	Function	Ref
*HIF1A*	Peripheral blood samples from 80 ApoE^-/-^ C57BL/6J male mice aged 4–6 weeks old and weighed 16–21 g (AS group included 60 mice and the sham group included 20 mice)	Upregulated	SMCs	USF1	-	Its knockdown inhibits the ox-LDL-induced atherosclerotic inflammation in SMCs by downregulating *ATF2* via binding USF1	[15]
*CDKN2B-AS1*	Venous blood samples from 15 patients with CHD and 15 healthy controls	Downregulated	VSMCs	*miR-126-5p*	PI3K/Akt signaling pathway	Inhibits VSMC proliferation and inflammation and boosts apoptosis through targeting *miR-126-5p*	[24]
*Kcnq1ot1*	200–250 g male SD rats (AMI group included 35 mice and sham group included 35 mice)	Upregulated	H9c2	*miR-466k* and *miR-466i-5p*	-	Triggers cardiomyocyte apoptosis by elevating Tead1 via sponging *miR-466k* and *miR-466i-5p*	[25]
*Mirt2*	20 adult male SD rats aged 12–14 weeks (AMI group included 10 mice and sham group included 10 mice)	Upregulated	H9c2	*miR-764*	-	Modulates cardiomyocyte injury by regulating the miR-764/PDK1 axis	[26]
*LINC00261*	100 male C57B/L6 mice aged 8–10 weeks old and weighed 18–25 g (MI group included 80 mice and the sham group included 20 mice)	Upregulated	H9c2	*miR-522-3p*	-	Its knockdown inhibits cardiomyocytes apoptosis by targeting *miR-522-3p*	[27]
*MIAT*	24 male C57BL6/J mice (weight 17–25 g) were established as ischemia/reperfusion (I/R) models	Upregulated	HCM	*miR-181a-5p*	JAK2/STAT3 signaling pathway	Its knockdown inhibits cell apoptosis and inflammation by regulating the JAK2/STAT3 signaling pathway via targeting *miR-181a-5p*	[28]
*MIAT*	70 male SPF C57BL/6J mice (MI group included 60 mice and sham group included 10 mice)	Upregulated	HL-1	*miR-10a-5p*	-	Promotes cardiomyocyte apoptosis by regulating the miR-10a-5p/EGR2 axis	[29]
*MALAT1*	32 male SD mice aged 14 weeks established as acute myocardial infarction (AMI) models	Upregulated	HL-1	*miR-125b-5p*	-	Promotes cardiomyocyte apoptosis through regulating miR-125b-5p/NLRC5 axis	[30]
*SNHG14*	Clean C57BL/6J mice and ApoE^-/-^ mice established as AS models	Downregulated	HA-VSMC	*miR-19a-3p*	-	Its overexpression promotes proliferation and inhibits apoptosis of VSMCs by regulating the miR-19a-3p/ROR α axis	[31]
*NR_045363*	Neonatal ICR/CD1 mice established as myocardial infarction (MI) models	Upregulated	HA-VSMC	-	p53 signaling pathway	Alleviates cardiomyocyte apoptosis by inhibiting the p53 pathway	[32]
*GAS5*	Mice (MI group included 15 mice and sham group included 15 mice)	Downregulated	H9c2	*miR-21*	PI3K/AKT signaling pathway	Its overexpression promotes cardiomyocyte apoptosis and inhibits cardiomyocyte proliferation by elevating *PDCD4* via sponging *miR-21*	[33]
*FOXC2-AS1*	Samples from 35 patients with AS and 35 healthy controls	Upregulated	HVSMC	*miR-1253*	-	Promotes proliferation and inhibit apoptosis of VSMCs via targeting the miR-1253/FOXF1 signaling axis	[34]

Abbreviations: HVSMCs, human vascular smooth muscle cells; VSMCs, vascular smooth muscle cells; HA-VSMC, human aortic vascular smooth muscle cells; HCM, human cardiomyocytes.

Compared with the control group, lncRNA *MHRT* levels in the border region of myocardial infarction in AMI mice were significantly increased, and *miR-3185* was proven to be a direct target gene of *MHRT* regulating fibrosis. Overexpressed lncRNA *MHRT* promotes myocardial fibrosis after myocardial infarction by negatively regulating *miR-3185* and increasing TGF-β1-induced proliferation of myocardial fibroblasts and intracellular deposition of collagen fibers Ⅰ and Ⅲ. Therefore, *MHRT* may be a therapeutic target for myocardial fibrosis [37]. Zou *et al*. [38] found that the migration and proliferation of cardiac fibroblasts (CFs) in lncRNA *ZFAS1* knockdown mice were significantly inhibited after hypoxia treatment, and cardiac function was also improved. The Wnt/β-catenin signaling pathway is closely related to the pathological mechanism of myocardial infarction. This study further confirmed that inhibition of the Wnt/β-catenin signaling pathway can reverse the effects of shZFAS1 on cardiac fibroblasts and cardiac function, revealing a potential regulatory network. In AMI mouse heart tissue and Ang Ⅱ-induced CFs, lncRNA *SNHG7* gene knockout can significantly reduce Ang Ⅱ-induced apoptosis, collagen synthesis, and inflammatory responses of CFs. Therefore, lncRNA *SNHG7* depletion exerts its functions through binding *miR-455-3p*, thus playing a protective role via regulating the platelet-activating factor receptor [39]. Luo *et al*. [40] found that lncRNA *554* at least partially regulates collagen synthesis and myocardial fibrosis after myocardial infarction by activating the TGF-β1 signaling pathway. Although its downstream target molecules still need to be further studied, it is clear that the knockdown of the lncRNA *554* gene will become a target for inhibiting cardiac fibrosis. Table 3 (Ref. [37, 38, 39, 40, 41, 42]) summarizes the studies between lncRNA and cardiac fibrosis in CAD. These results show the role of lncRNAs in CFs. The increased proliferation of CFs and deposition of extracellular matrix proteins have been described as cardiac fibrosis, which severely affects the prognosis of CAD [43]. One study suggests that the classical TGF-β and WNT signaling pathways display information crosstalk that appears to regulate the fibrosis process in CAD [44]. The signaling pathways listed in Table 3 include only TGF-β and WNT signaling pathways involved in regulating myocardial fibrosis. Whether other signaling pathways are potentially involved through these pathways is unknown. The specific mechanisms by which lncRNA’s impact cardiac fibrosis by regulating these two signaling pathways needs to be further determined.

**Table 3.** **CAD-related lncRNAs whose function has been assessed in CFs**.

LncRNA	Sample	Expression pattern	Assessed cell lines	Gene/protein interactions	Signaling pathway	Function	Ref
*MHRT*	Healthy male C57 BL/6 mice (20–25 g) were established as MI model	Upregulated	CFs	*miR-3185*	-	Its knockdown inhibits CFs collagen production and proliferation	[37]
*ZFAS1*	Male Wistar rats (weighting 240–260 g) were established as MI model	Upregulated	CFs	-	Wnt/β-catenin signaling pathway	Its knockdown increases the proliferation, migration, and invasion of CFs by regulating the Wnt/β-catenin signaling pathway	[38]
*SNHG7*	10 male C57BL/6J mice aged 10 weeks (23–25 g) (MI group included 5 mice and sham group included 5 mice)	Upregulated	CFs	*miR-455-3p*	-	Its knockdown inhibits Ang-II-induced apoptosis, collagen synthesis, and inflammation in CFs by regulating miR-455-3p/PTAFR axis	[39]
*lncRNA 554*	Male C57BL/6 mice aged 8–10 weeks (20–30 g) were established as MI model	Upregulated	CFs	-	TGF-β1 signaling pathway	Its knockdown attenuates cardiac fibrosis by regulating the TGF-β1 signaling pathway	[40]
*Ang362*	60 male SD mice weighing 250–300 g (control group included 20 mice, sham-operated group included 20 mice, MI group included 20 mice)	Upregulated	CFs	Smad7	-	Contributes to cardiac fibrosis post-MI by inhibiting Smad7 expression	[41]
*MALAT1*	Male C57BL/6 mice aged 12–16 weeks (weighting 21–25 g) were established as MI model	Upregulated	NMCFs	*miR-145*	TGF‐β1 signaling pathway	Its knockdown attenuates cardiac fibrosis and alleviates AngII‐induced cell proliferation, collagen production, and myofibroblast transdifferentiation by regulating the TGF‐β1 signaling pathway via *miR‐145*	[42]

Abbreviations: CFs, cardiac fibroblasts; PTAFR, platelet-activating factor receptor; NMCFs, neonatal mouse cardiac fibroblasts.

## 3. Diagnostic/Prognostic Significance of LncRNAs in CAD

Changes in circulating lncRNA expression levels in patients with CAD make them potential biomarkers for diagnosis and prognosis. Liu *et al*. [45] found that compared with a normal coronary artery group, there were 98 differentially expressed lncRNAs in peripheral blood mononuclear cells of unstable angina patients. A ROC curve was used to reflect the relationship between the sensitivity and specificity of these lncRNAs. The AUC, which was between 0.1 and 1, can be used to directly evaluate the diagnostic value of the lncRNA; the larger the value, the greater the diagnostic potential.

**Fig. 1.**
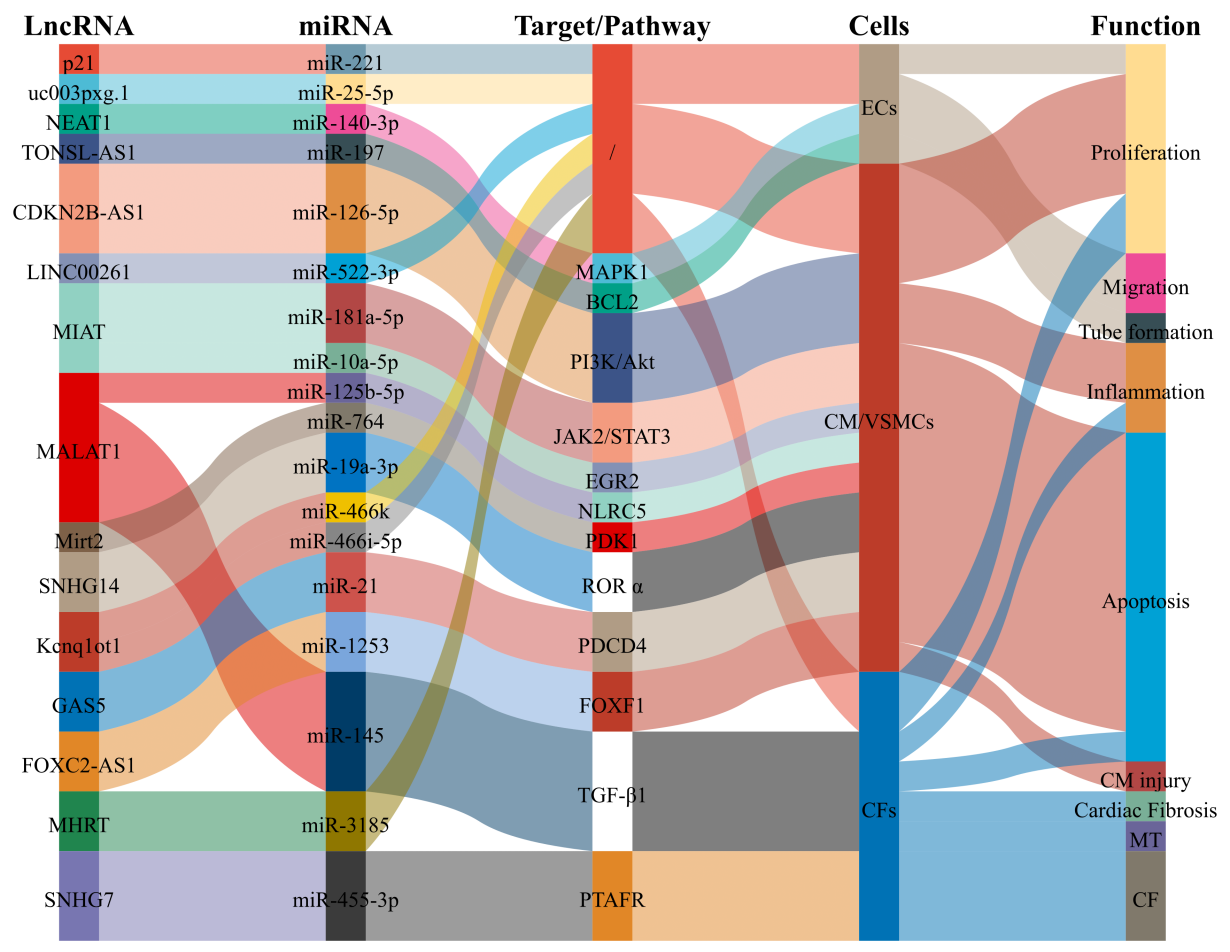
**LncRNAs as biomarkers of CAD participate in pathological processes and then affect the occurrence and development of CAD**. Abbreviations: ECs, endothelial cells; VSMCs, vascular smooth muscle cells; CM, cardiomyocyte; CF, cardiac fibrosis.

Among 98 differentially expressed lncRNAs, the AUC values of *MALAT1* and *LNC_000226* were 0.81 and 0.799, respectively. Both have very high diagnostic values, distinguishing unstable angina patients from the normal group. However, the specificity of *LNC_000226* was not very high [45]. In addition, compared with healthy subjects, lncRNA* MIAT* was significantly overexpressed in the peripheral blood of CAD patients, which is likely to be an important biomarker for the diagnosis of CAD. LncRNA *MIAT* had an AUC of 0.908 and sensitivity and specificity of 0.700 and 0.714, respectively, and is one of the independent risk factors for CAD patients [46]. Studies have shown that the inflammatory mediators tumor necrosis factor-alpha (TNF-α), monocyte chemotactic protein-1 (MCP-1), vascular cell adhesion molecule-1 (VCAM-1), intercellular adhesion molecule-1 (ICAM-1), and interleukin-6 (IL-6) positively correlate with risk stratification in patients with coronary heart disease, but these inflammatory mediators were negatively correlated with plasma lncRNA *FA2H-2* levels. LncRNA *FA2H-2*, as an independent risk factor for coronary heart disease, was expressed with an area under the ROC curve of 0.834 and sensitivity and specificity of 0.85 and 0.82, respectively. Therefore, lncRNA *FA2H-2* is likely to distinguish the control group from the CHD group [47]. The increased number of coronary lesion vessels was an independent risk factor for poor prognosis in patients with CAD. ROC curve analysis indicated that the AUC of lncRNA FGF9-associated factor (FAF) had a prognostic value in CAD of 0.916 in addition to a high diagnostic value of 0.935. Additionally, compared with controls with no major adverse cardiac events, the expression of *FAF* in patients with major adverse cardiac events group was lower [48]. The diagnostic/prognostic effects of lncRNAs in CAD are summarized in Table 4 (Ref. [22, 45, 46, 47, 48, 49, 50, 51, 52, 53]). All the lncRNAs listed in the table have high diagnostic values; however, the specificity and sensitivity of some lncRNAs have not been determined. The biomarkers of CAD lncRNAs and their mechanisms of action are summarized in Fig. 1.

**Table 4.** **Diagnostic/prognostic significance of lncRNAs in CAD**.

LncRNAs	Expression pattern	Sample	Diagnostic/Prognostic role	ROC curve analysis			Ref
				Sensitivity	Specificity	AUC	
*FA2H-2*	Downregulated	Blood samples from 316 patients with coronary heart disease	Diagnostic biomarker	0.850	0.820	0.834	[47]
*MALAT1*	Upregulated	Blood samples from 140 patients with coronary heart disease and 90 controls	Distinguishing CHD patients from normal subjects	-	-	0.837	[49]
*FAF*	Downregulated	A serum sample from patients with 97 coronary heart disease and 97 controls	Diagnostic and prognostic biomarker	-	-	0.935/0.916	[48]
*ENST00000416361*	Upregulated	Blood samples from 187 patients with CAD and 150 controls	Diagnostic biomarker	-	-	0.790	[50]
*CASC11*	Downregulated	Plasma samples from 82 patients with CAD and 82 controls	Diagnostic biomarker	-	-	0.900	[22]
*LNC_000226*	Upregulated	PBMC from 44 patients with UA and 44 NCA	Diagnostic biomarker	0.957	0.587	0.810	[45]
*MALAT1*	Upregulated	PBMC from 44 patients with UA and 46 NCA	Diagnostic biomarker	0.705	0.848	0.799	[45]
*MIAT*	Upregulated	Peripheral venous blood samples from 155 patients with CAD and 76 controls	Diagnostic biomarker	0.700	0.714	0.908	[46]
*NEAT1*	Upregulated	Peripheral venous blood samples from 47 patients with STEMI and 24 patients with UA and 27 controls	Distinguishing STEMI patients from normal subjects and UA patients	0.638	0.882	0.822	[51]
*SOCS2-AS1*	Downregulated	Blood samples from 111 patients with CAD and 48 patients with mild coronary artery stenosis (mCAS) and 68 controls	Discriminating CAD patients from controls	0.714	0.634	0.704	[52]
*HULC*	Downregulated	Blood samples from 50 patients with CAD and 50 normal subjects	Discriminating CAD patients from normal subjects	-	-	0.900	[53]
*DICER1-AS1*	Downregulated	Blood samples from 50 patients with CAD and 50 normal subjects	Discriminating CAD patients from normal subjects	-	-	0.870	[53]

Abbreviations: UA, unstable angina; STEMI, ST-elevation myocardial infarction; PBMC, peripheral blood mononuclear cells; ROC, receiver operating characteristic; AUC, area under the curve; NCA, normal coronary artery.

## 4. Conclusions and Perspectives

Our review has found that most lncRNAs act as ceRNAs, competing with downstream target miRNAs to regulate the pathophysiological process of CAD through different mechanisms, such as regulation of vascular endothelial cells, regulation of vascular smooth muscle cell activity, regulation of myocardial cell proliferation and apoptosis, collagen fiber production, and myocardial fibroblast function. The JAK1/STAT3 signaling pathway, AKT signaling pathway, and other signaling pathways are all lncRNA signaling pathways involved in CAD. lncRNAs affect different aspects and different targets in this process. For example, lncRNA *MIAT* regulates the expression of *miR-181a-5p* and *miR-10a-5p*; both are involved in cardiomyocyte apoptosis. The knockdown of both lncRNA *MALAT1* and lncRNA *554* can inhibit myocardial fibrosis through the TGF-β1 signaling pathway. This suggests that there may be a rich regulatory network linking lncRNAs with CAD. Most importantly, differences in circulating lncRNA expression levels can also be used to distinguish healthy individuals from patients with CAD, as well as ST-elevation myocardial infarction and UA patients, serving as markers for diagnosis and prediction of disease progression. The stability of lncRNAs and their easy extraction from serum and body fluids make them easier to detect. Increased myocardial fibrosis (MF) following a myocardial infarction is a major cause of heart failure (HF). Studies have shown that several lncRNAs are differentially expressed in the transition from MI to MF to HF. This may have the potential for predicting disease progression and prognosis in patients with an MI, but the detailed molecular and pathological mechanisms involved in the progression from MI-MF-HF have not been thoroughly validated.

The mortality rate in CAD remains high, largely because of the lack of effective drugs to prevent or decrease myocardial ischemic necrosis in clinical practice. LncRNAs may become the prototype to create effective drugs. In-depth exploration of the complex splicing process, differential transcriptional processing, differential intracellular expression, and subcellular localization of lncRNAs will contribute to the understanding of the pathogenesis and regulatory mechanism of CAD and lead to improvements in the clinical diagnosis and treatment of CAD. In summary, lncRNAs are involved in regulating many aspects of the pathogenesis of CAD and can be used as specific/sensitive markers for this disease. The diagnostic/prognostic/therapeutic role of lncRNAs in CAD will need to be explored in future studies.
